# Meta-Analysis of Transcriptome-Wide Association Studies across 13 Brain Tissues Identified Novel Clusters of Genes Associated with Nicotine Addiction

**DOI:** 10.3390/genes13010037

**Published:** 2021-12-23

**Authors:** Zhenyao Ye, Chen Mo, Hongjie Ke, Qi Yan, Chixiang Chen, Peter Kochunov, L. Elliot Hong, Braxton D. Mitchell, Shuo Chen, Tianzhou Ma

**Affiliations:** 1 Maryland Psychiatric Research Center, Department of Psychiatry, School of Medicine, University of Maryland, Baltimore, MD 21201, USA; zye@som.umaryland.edu (Z.Y.); chen.mo@som.umaryland.edu (C.M.); PKochunov@som.umaryland.edu (P.K.); Ehong@som.umaryland.edu (L.E.H.); 2 Division of Biostatistics and Bioinformatics, Department of Epidemiology and Public Health, School of Medicine, University of Maryland, Baltimore, MD 21201, USA; Chixiang.Chen@som.umaryland.edu; 3 Department of Epidemiology and Biostatistics, School of Public Health, University of Maryland, College Park, MD 20742, USA; kehj@umd.edu; 4 Irving Medical Center, Department of Obstetrics & Gynecology, Columbia University, New York, NY 10032, USA; qy2253@cumc.columbia.edu; 5 Department of Medicine, University of Maryland School of Medicine, Baltimore, MD 21201, USA; bmitchel@som.umaryland.edu

**Keywords:** genome-wide association study, transcriptome-wide association study, meta-analysis, expression quantitative trait loci, nicotine addiction

## Abstract

Genome-wide association studies (GWAS) have identified and reproduced thousands of diseases associated loci, but many of them are not directly interpretable due to the strong linkage disequilibrium among variants. Transcriptome-wide association studies (TWAS) incorporated expression quantitative trait loci (eQTL) cohorts as a reference panel to detect associations with the phenotype at the gene level and have been gaining popularity in recent years. For nicotine addiction, several important susceptible genetic variants were identified by GWAS, but TWAS that detected genes associated with nicotine addiction and unveiled the underlying molecular mechanism were still lacking. In this study, we used eQTL data from the Genotype-Tissue Expression (GTEx) consortium as a reference panel to conduct tissue-specific TWAS on cigarettes per day (CPD) over thirteen brain tissues in two large cohorts: UK Biobank (UKBB; number of participants (N) = 142,202) and the GWAS & Sequencing Consortium of Alcohol and Nicotine use (GSCAN; N = 143,210), then meta-analyzing the results across tissues while considering the heterogeneity across tissues. We identified three major clusters of genes with different meta-patterns across tissues consistent in both cohorts, including homogenous genes associated with CPD in all brain tissues; partially homogeneous genes associated with CPD in cortex, cerebellum, and hippocampus tissues; and, lastly, the tissue-specific genes associated with CPD in only a few specific brain tissues. Downstream enrichment analyses on each gene cluster identified unique biological pathways associated with CPD and provided important biological insights into the regulatory mechanism of nicotine dependence in the brain.

## 1. Introduction

The past decade has witnessed an explosion in genome-wide association studies (GWAS) research, which identified thousands of robust, reproducible genetic risk variants associated with complex diseases and traits [1,2]. These findings have contributed to a better understanding of disease biology and the relative roles of genes vs. environment in disease risk [3,4]. However, the loci identified by GWAS are not directly interpretable due to the strong linkage disequilibrium (LD) that obscures the causal variants, and GWAS data alone can hardly determine the causal genes and the underlying regulatory mechanism [5]. To fill this gap, transcriptome-wide association studies (TWAS) are developed to utilize expression quantitative trait loci (eQTL) cohorts (e.g., Genotype-Tissue Expression (GTEx) [6]), which include both genotype and gene expression data as a reference panel to infer association with a trait at the gene level [7]. In short, TWAS involve training a predictive model of expression from the genotype in the reference panel, then using the trained model to predict the expression in the GWAS data, which are used to find the genes associated with the trait [7,8]. Various statistical methods and computational tools for implementing TWAS have been developed to date [9,10]. Since gene expression and eQTL regulation are tissue-dependent, TWAS are usually conducted in a tissue-specific manner. For example, PrediXcan [8] is the first ever TWAS tool that leverages the single nucleotide polymorphism (SNP)-gene associations identified in a single tissue to infer gene-trait associations. S-PrediXcan [11] is an extension of PrediXcan that takes GWAS summary statistics as the input. Considering the similarity in transcription regulation across tissues, MultiXcan [12] and UTMOST [13] fit models to integrate the information of SNP-gene associations across multiple tissues to infer the gene-trait associations.

To date, a lot of genetic research has revealed the important role of genetic factors on nicotine dependence [14,15]. For example, GWAS have identified susceptible genetic variants located in the nicotinic acetylcholine receptors (nAChRs) [16], metabolic enzyme encoded gene *CYP2A6* [17], and lung-specific genes *TENM2* [18] associated with nicotine addiction. However, how these genetic compositions contribute to human nicotine dependence behaviors and the underlying molecular regulatory mechanism in the brain remained largely unknown. Palmer et al. [19] conducted a cross-species TWAS analysis of tobacco consumption by integrating human GWAS data from UK Biobank (UKBB) and messenger ribonucleic acid (mRNA) expression references from the brains of multiple animal species and identified 10 homologous genes associated with the cigarettes per day (CPD) in different animal models, to illustrate the genetic mechanisms of human tobacco consumption. However, the heterogeneity among eQTL datasets and tissues-dependent nature of transcription regulation have impeded the ability of TWAS to provide further insights into the genetic basis of diseases [20].

Meta-analysis is a set of powerful statistical tools, which combines multiple related studies for various biological purposes and has gained popularity in both GWAS and omics research in recent years [21,22]. Traditional meta-analysis methods, such as Fisher’s and Stouffer’s, combine *p*-values from multiple studies without further exploring the association patterns across studies [23,24]. New meta-analysis methods have been proposed to account for the heterogeneity across studies and categorize biomarkers (e.g., genes) by their cross-study patterns while combining the studies [25,26,27]. In this study, we performed tissue-specific TWAS of nicotine addiction (measured by CPD) for 13 brain tissues based on the GWAS data from UKBB [28] and GWAS & Sequencing Consortium of Alcohol and Nicotine use (GSCAN) [29], using eQTL cohorts from GTEx (version 8) [6] as the reference panel. We then conducted meta-analysis of the TWAS results while considering the heterogeneity across tissues and clustered the nicotine-addiction-associated genes by their cross-tissue patterns. Such a procedure was shown to be more powerful than a multi-tissue TWAS tool (e.g., S-MultiXcan) and detected novel clusters of genes with different meta-patterns across brain tissues. Downstream enrichment analysis on the different clusters of genes identified important nicotine-addiction-related pathways in different brain tissues and provided more insights into the molecular regulatory mechanisms underlying nicotine dependence inside the brain.

## 2. Materials and Methods

### 2.1. Study Cohorts

In this study, we performed TWAS analysis on two large cohorts that include both genotype and nicotine addiction phenotype data:

(1) UK Biobank (UKBB): a large prospective study that recruited ~500,000 participants aged between 40–69 years in 2006–2010 in 22 assessment centers throughout the UK and collected abundant phenotypic and genomic data [28]. For this study, we included number of participants (N) = 142,202 individuals with white ethnicity backgrounds (British, Irish, and any other white background) that had both genotype- and nicotine-dependence-related smoking phenotypic data available.
(2) GWAS & Sequencing Consortium of Alcohol and Nicotine use (GSCAN): a meta-analysis of up to 35 GWAS cohorts of European ancestry including around 1.2 million individuals (depending on traits) [29]. Smoking-related phenotypes in the GSCAN were self-reported responses gathered by multiple teleconferences [29]. We used the GSCAN data excluding UKBB and 23andMe (“Minus23andMeUKBB” with N = 143,210) as a validation cohort [https://conservancy.umn.edu/handle/11299/201564, accessed on 16 November 2021]. For GSCAN, only meta-analyzed GWAS summary data were available.

### 2.2. Nicotine Dependence Related Smoking Phenotype

Cigarettes per day (CPD) is one of the well-known traits related to nicotine addiction and is widely used in many published studies [30,31]. We used CPD as the phenotype of interest in our study. For the UKBB cohort, CPD was defined as the average number of cigarettes smoked per day by participants who were either current or past smokers, using phenotype codes 2887 (number of cigarettes previously smoked daily), 3456 (number of cigarettes currently smoked daily) and 6183 (number of cigarettes previously smoked daily (current cigar/pipe smokers)). The CPD values of participants who smoked less than one cigarette per day were recoded to 0; and CPD values of those who smoked more than 60 cigarettes per day were recoded to 60. CPD was denoted as CigDay in GSCAN cohort. The detailed data processing procedure of the CigDay can be found in Liu et al. [29].

### 2.3. Reference Panel

The reference panel of the eQTL cohort used to perform TWAS analysis was obtained from the Genotype-Tissue Expression (GTEx) project (version 8) [6]. It included both the genotype data of 838 donors of mainly European ancestry and the gene expression data of these donors in 13 brain tissues, including amygdala, anterior cingulate cortex (BA24), caudate (basal ganglia), cerebellum, cerebellar hemisphere, cortex, frontal cortex (BA9), hippocampus, hypothalamus, nucleus accumbens (basal ganglia), putamen (basal ganglia), spinal cord (cervical c-1), and substantia nigra. The single-tissue predicted weights and single-/across-tissue LD reference files from GTEx used in S-PrediXcan [11] and S-MultiXcan [12] were provided by PredictDB (https://hakyimlab.org/post/2020/01/07/predictdb-transcriptome-prediction-model-repository/, accessed on 16 November 2021) for use in our study.

### 2.4. TWAS Analysis

**Figure 1:**
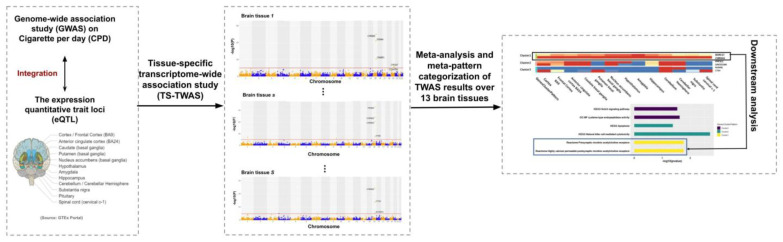
Study scheme. We integrated genome-wide association studies (GWAS) summary statistics with the quantitative trait loci (eQTL) reference panel from Genotype-Tissue Expression (GTEx) to conduct tissue-specific transcriptome-wide association studies (TS-TWAS) analysis for each of the 13 brain tissues using S-PrediXcan. We then performed meta-analysis of the TS-TWAS results across tissues using adaptively weighted Fisher’s (AW-Fisher’s) method and clustered the genes by their meta-patterns across tissues. We additionally performed downstream analysis (e.g., pathway enrichment analysis) to each category of genes with a unique meta-pattern.

In this study, we first conducted tissue-specific TWAS (TS-TWAS) of CPD for each of the 13 brain tissues by combining GWAS data with the eQTL reference panel and then performed meta-analysis to combine the TS-TWAS results and categorize the CPD associated genes by their meta-patterns across the tissues (Figure 1). Below, we describe the two steps of our analysis in detail.

#### 2.4.1. Tissue-Specific TWAS

In the first step, we conducted TS-TWAS for each of the 13 brain tissues using S-PrediXcan [11]. For UKBB, we first performed GWAS on CPD of 142,202 participants (Mean Age = 57.57 (7.83); 48.12% are Female) using PLINK (version 1.9, www.cog-genomics.org/plink/1.9/, accessed on 16 November 2021) [32] under an additive genetic model. We performed quality control and removed variants with a minor allele frequency below 0.01, Hardy-Weinberg equilibrium *p*-value below 0.001, and missing genotype rate at 5%, and excluded individuals with more than 2% missing genotypes. The UKBB cohort had relatively low prevalence of self-reported health conditions (e.g., brain injury, neuropsychiatric complications) and light-to-moderate consumptions of alcohol and cannabis (Tables S1 and S2); thus, we did not further consider additional exclusion criteria. The analysis was adjusted by the following variables: sex, age, body mass index (BMI), genotyping chip type, and top ten principal components of population admixture generated from PLINK (version 2.0, www.cog-genomics.org/plink/2.0/, accessed on 16 November 2021) [32]. For GSCAN, a GWAS summary on CPD was directly obtained from the University of Minnesota library [29]. We integrated GWAS summary statistics of both cohorts with the pre-trained prediction models over 13 brain tissues to obtain TS-TWAS results using S-PrediXcan [11], an extension of PrediXcan [8] that used only summary level GWAS statistics to estimate a Z-score of association between gene expression and trait. The tissue-specific Z-score for the g-th gene in s-th tissue can be estimated as follows:

$$z_{gs}\approx \sum_{l\in Model_{gs}}w_{lgs}\frac{{\hat{\sigma }}_{l}}{{\hat{\sigma }}_{gs}}\frac{{\hat{\beta }}_{l}}{se\left({\hat{\beta }}_{l}\right)}$$

where $Model_{gs}$ is the pre-trained prediction model from the GTEx reference panel, consisting of SNPs used to predict the gene expression for gth gene in sth tissue, $w_{lgs}$ is the predicted weight of l-th SNP on the g-th gene in s-th tissue in the pre-trained prediction model, directly obtained from PredictDB. ${\hat{\beta }}_{l}$ is the GWAS estimate for l-th SNP; $se\left({\hat{\beta }}_{l}\right)$ is GWAS standard error of ${\hat{\beta }}_{l}$; ${\hat{\sigma }}_{l}$ is the variance of l-th SNP; and ${\hat{\sigma }}_{gs}$ is the variance of the predicted expression for g-th gene in s-th tissue. The SNP variance term ($\frac{{\hat{\sigma }}_{l}}{{\hat{\sigma }}_{gs}}$) calculated from 1000 Genomes data was also obtained from PredictDB. We computed the *p*-value for the g-th gene in s-th tissue as $p_{\mathrm{gs}}=2(1-\Phi \left(z_{gs}\right)$), where Φ(.) is the cumulative density function of standard normal distribution.

#### 2.4.2. Meta-Analysis of TS-TWAS over 13 Brain Tissues and Downstream Analysis

Adaptively weighted (AW)-Fisher’s method [25] is a meta-analysis method extending the conventional Fisher’s method that combines *p*-values from multiple studies while taking the study to study heterogeneity into account. In this paper, we treated different brain tissues as studies and applied AW-Fisher’s method to meta-analyze the TS-TWAS results from all S = 13 brain tissues. The null hypothesis in meta-analysis is commonly considered as

$$H_{0}:\theta_{g1}=\ldots =\theta_{\mathrm{gS}}=0,$$

where $\theta_{\mathrm{gs}}$ is the gene effect of g-th gene in the s-th tissue. For an alternative hypothesis, we aimed to detect genes associated with CPD in at least one tissue, i.e., $H_{a}:\theta_{\mathrm{gs}}\neq 0$ for some 1 ≤ s ≤ S. For AW-Fisher’s method,

$$U_{g}\left(\omega_{g}\right)=-\overset{S}{\underset{s=1}{\sum }}\omega_{gs}\log\left(p_{\mathrm{gs}}\right),$$

where $p_{\mathrm{gs}}$ is the *p*-value of the g-th gene in the s-th tissue from TS-TWAS, $\omega_{gs}$ is the 0–1 binary weight assigned to the s-th tissue, and $\omega_{g}=\left(\omega_{g1},\ldots ,\omega_{\mathrm{gS}}\right)$. For a specific $\omega_{g}$, the *p*-value of the observed weighted statistic $p_{U}\left(u_{g}\left(\omega_{g}\right)\right)\;$ under the null hypothesis can be obtained via permutation. The AW-Fisher’s statistic was defined as the minimal *p*-value among all possible weights. For inference, there is no closed-form distribution for AW-Fisher’s statistics under the null, so permutation tests and importance sampling are used to obtain the *p*-values $p_{g}^{\mathrm{AW}}$ and control the false discovery rate (FDR). More details can be found in the original AW-Fisher paper [25].

After meta-analysis, we focused on genes passing an FDR threshold of 0.05 in both cohorts (i.e., take the intersection) and performed downstream analysis. We categorized the genes by their meta-patterns across brain tissues using hierarchical clustering with Ward linkage on −log10$\left(p_{\mathrm{gs}}\right)$. For each category of genes, we further performed pathway enrichment analysis using three popular pathways datasets: Gene Ontology (GO) [33], Kyoto Encyclopedia of Genes and Genomes (KEGG) [34], and Reactome [35]. Top enriched pathways (e.g., Fisher’s exact test *p*-value < 0.05) helped us understand the unique functions for each category of genes associated with nicotine addiction but with different cross-tissue patterns. We also applied the S-MultiXcan method [12] for multi-tissue TWAS analysis across 13 brain tissues as a comparison.

## 3. Results

**Figure 2:**
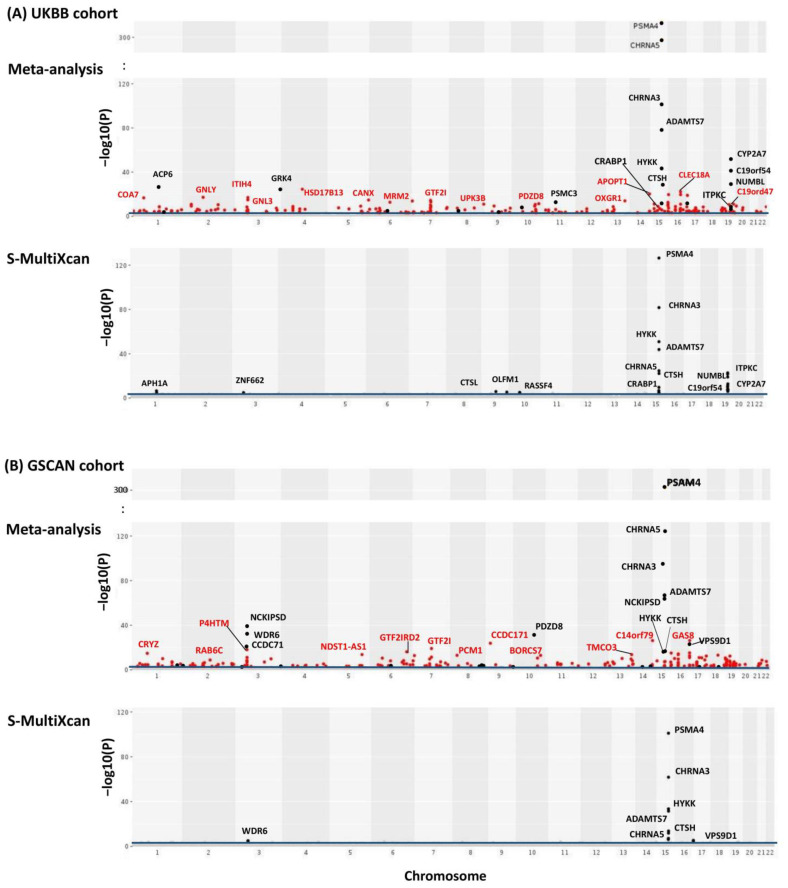
Manhattan plots of meta-analysis of TS-TWAS results across all 13 brain tissues for both UKBB (**A**) and GSCAN (**B**). *Y*-axis is the −log10($p_{g}^{AW}$) from AW-Fisher. Results from S-MultiXcan are used for comparison. The blue line indicates an FDR cutoff of 0.05. Genes detected by meta-analysis but not by S-MultiXcan were highlighted in red and genes passing the Bonferroni cutoff (i.e., *p* < 0.05/#genes) were labeled.

We first performed TS-TWAS and then meta-analyzed the TS-TWAS results over 13 brain tissues by AW-Fisher’s method. The meta-analysis of TS-TWAS identified 48 genes significantly associated with CPD at FDR < 0.05 common in both UKBB and GSCAN cohorts (Table 1). Comparing to S-MultiXcan and TS-TWAS, meta-analysis was overall more powerful in identifying more nicotine-addiction-associated genes (Table 1; Figure 2 highlighted in red, Figure S1), especially among genes with heterogeneous association patterns across tissues (Figure S1, Supplementary file 1). These included multiple nicotine-addiction-associated genes reported in previous studies [17,18,31]. We focused on the 48 genes at FDR < 0.05 for biomarker categorization and downstream analysis.

**Figure 3:**
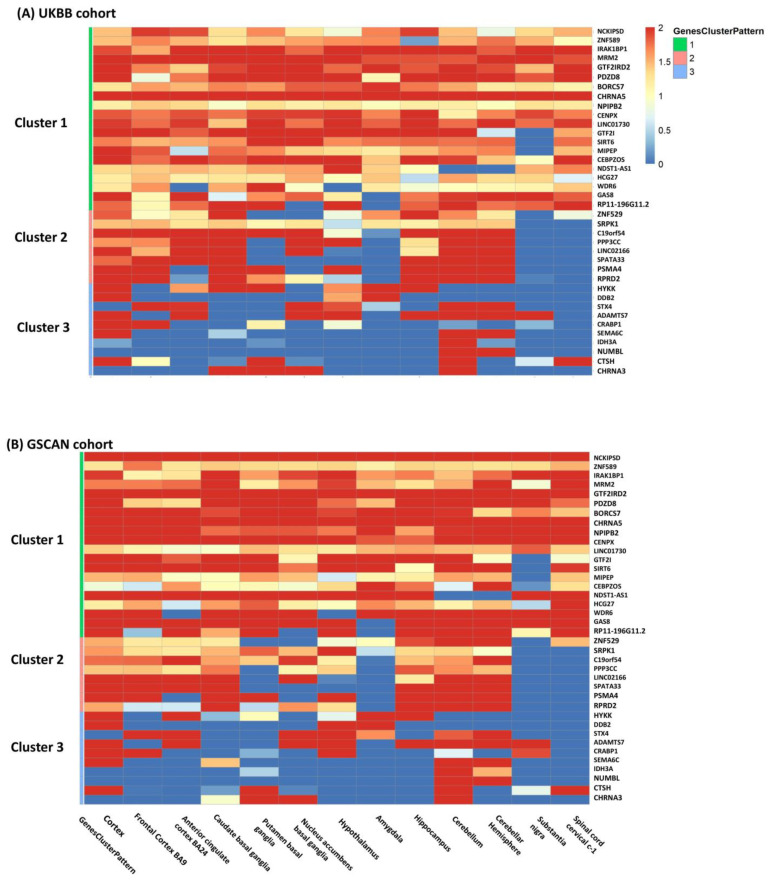
The heatmap included the 38 genes (Cluster 1: 20; Cluster 2: 8; Cluster 3: 10) with the same clustering patterns and passing meta-analysis FDR < 0.05 threshold in both cohorts and was colored by −log10($p_{\mathrm{gs}}$) of TS-TWAS in each brain tissue (on columns) from both cohorts (Panel (**A**) for UKBB and Panel (**B**) for GSCAN). In the rows, the genes were clustered into three categories common to two cohorts: cluster 1 was homogeneous genes, cluster 2 was partially homogeneous genes, and cluster 3 was tissue-specific or heterogeneous genes.

Gene categorization by meta-patterns identified three clusters of genes common to both cohorts (Figure 3): (i) homogeneous genes, which were associated with CPD in all 13 brain tissues; (ii) partially homogeneous genes, which were associated with CPD in a majority of tissues but not significant in the rest; and (iii) tissue-specific or heterogeneous genes, which had very unique association patterns in different tissues, reflecting a high degree of heterogeneity across tissues. The two cohorts had a large proportion of genes matched in each cluster (38 out of 48 genes in total; Table 1). The first cluster included 20 genes homogeneously associated with CPD in all brain tissues, including well-known smoking-related genes *CHRNA5*, *NCKIPSD*, and *SIRT6*. The second cluster consisted of 8 genes including *PSMA4* and *RPRD2*, which are highly expressed and associated with CPD in forebrain regions such as frontal cortex BA9, anterior cingulate BA24, and hindbrain regions including the cerebellum and cerebellar hemisphere. The third cluster included 10 heterogeneous genes only associated with CPD in very few specific brain tissues.

**Figure 4:**
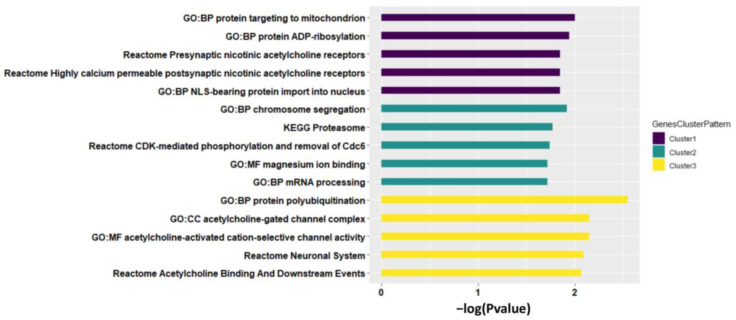
Top five pathways enriched by each cluster of genes identified, sorted by both *p*-value and pathway size. The *p*-value is from Fisher’s exact test.

We then performed pathway analysis to each cluster of identified genes. Cluster 1 of homogeneous genes (e.g., *CHRNA5*) was mainly enriched in pathways related to presynaptic and postsynaptic nicotinic acetylcholine receptors (Figure 4, see the full pathway analysis results in Supplementary file 2), that play versatile roles in neuronal apoptosis [36] and neurotransmission (e.g., Ca^2+^ signaling [37] and dopamine [38]). Cluster 2 of partially homogeneous genes (e.g., *PSMA4*) was enriched in pathways related to proteasomal activity (e.g., KEGG Proteasome), intercellular bivalent cations Mg^2+^ (e.g., GO:MF magnesium ion binding), and chromosome segregation (e.g., GO:BP chromosome segregation) that can be highly impacted by cigarette smoking to inhibit proteasomal activity, cause mental disorders disease, and induce segregation anomalies separately reported in previous studies [39,40,41]. Cluster 3 of heterogeneous genes (e.g., *CHRNA3*) was enriched in pathways GO:MF acetylcholine-activated cation-selective channel activity and GO:CC acetylcholine-gated channel complex. Genes with different meta-patterns across tissues were functionally specific that are worth further investigation in future studies.

## 4. Discussion

In this study, we used an eQTL reference panel from GTEx to conduct meta-analysis of TS-TWAS on nicotine addiction over 13 brain tissues in two large cohorts, UKBB and GSCAN. The meta-analysis was shown to be more powerful than the multi-tissue TWAS method implemented in S-MultiXcan [12], by detecting more nicotine addiction associated genes while accounting for the heterogeneity across multiple brain tissues. In addition to detecting more associated genes, gene categorization by meta-patterns identified three novel clusters of genes common to both cohorts, including 20 genes homogeneously associated in all brain tissues, 8 genes partially homogeneously associated mainly in the cortex and cerebrum, and 10 genes with tissue-specific association. Several well-known nicotine-addiction-associated genes, including *CHRNA5*, *PSMA4*, and *CHRNA3*, were identified and their cross-brain-tissue-association patterns were revealed. To the best of our knowledge, our study was the first comprehensive meta-analysis of TWAS on nicotine addiction across 13 major brain tissues, investigated and validated in two large-scale epidemiological cohorts (UKBB and GSCAN).

The first cluster of genes was enriched in pathways related to presynaptic and postsynaptic nicotinic acetylcholine receptors, as marked by the gene *CHRNA5*. Previous GWAS have identified multiple reproducible variants in *CHRNA5* [29,31], which were attributed to functions in both the enhancement and aversion of nicotine intake [42]. We further showed in our study that *CHRNA5* was highly expressed in all brain tissues, and its association with CPD was consistent throughout the brain. The second cluster was marked by the proteasome gene *PSMA4*. Its association with CPD was prominent in the frontal lobe (e.g., frontal cortex BA9 and anterior cingulate BA24), cerebellum/cerebellar hemisphere, and hippocampus tissues. The enriched biological pathways were driven by PSMA4, playing a central role in decreasing neuronal proteasome activity [43]. Another nicotinic acetylcholine receptor gene, *CHRNA3*, was observed in the third cluster with tissue-specific association with CPD only in putamen basal ganglia and nucleus accubens basal ganglia. The elevated dopamine activity in the basal ganglia region of cigarette smokers has been identified for the enriched pathways driven by *CHRNA3* in the previous studies [44,45]. These findings showed the strength of our comprehensive meta-analysis of TWAS on CPD that identified novel clusters of genes with unique meta-patterns across tissues, inferring different biological function.

TWAS are getting popular over recent years as a promising complement to GWAS by incorporating the functional annotation information and analyzing association with the trait at the gene level. Despite the foreseen success, most TWAS methods to date are tissue specific and ignore the similarity in transcription regulation across tissues, usually having a limited effective sample size and, thus, being underpowered [13]. Our study performed the meta-analysis of TS-TWAS across 13 brain tissues on nicotine addiction and categorized the identified genes by their meta-patterns across tissues. Such a meta-analytical framework can be applied to study other addictions (such as alcohol [46] and cannabis use [47]) and disorders strongly associated with altered brain structure as well as explore potential distinct gene clusters in specific brain regions. In addition, it is widely applicable to analyze other traits through the targeting of other tissues and eQTL reference panels (e.g., blood [48] and lung [49]). One of the main challenges of the TWAS approach is that it is difficult to prioritize causal genes due to co-regulation [7]. A Mendelian randomization framework incorporated into the TWAS for identification of putative causal inference needs to be conducted to carve out this issue [10,50,51]. Further studies, such as the application of fine-mapping methods (e.g., FOCUS [52]), will be needed to confirm our meta-TWAS results and to distinguish the causal genes for nicotine addiction, which can improve our understanding of the genetic basis of brain-related disorders.

Lastly, our results are based on analyzing two big cohorts, UKBB and GSCAN, so are more generalizable to the general population. Though the UKBB cohort consists of mainly healthier participants, health volunteer selection bias will not largely affect the credibility of results, as has been noted in several previous studies [53,54,55]. Future studies can be conducted to correct this health volunteer effect, such as propensity scores modification [56] and weighted analysis [57], to further improve the generalizability of the study results.

## Figures and Tables

**Table 1:** Summary of the number of cigarettes per day (CPD)-associated genes detected by meta-analysis and S-MultiXcan as well as in each category of unique meta-pattern in both UK Biobank (UKBB) and GWAS & Sequencing Consortium of Alcohol and Nicotine use (GSCAN) cohorts and their intersection.

Cohort		UKBB	GSCAN	Intersection
S-MultiXcan (FDR < 0.05)		60	13	8
Meta-analysis by AW-Fisher’s method (FDR < 0.05)		245	217	48
Meta-pattern categorization (48 genes in intersection at FDR < 0.05)	Cluster 1 (homogeneous genes)	24	22	20
	Cluster 2 (partially homogeneous genes)	12	12	8
	Cluster 3 (tissue-specific or heterogeneous genes)	12	14	10

## Data Availability

The raw genetic and phenotypic data used in the current study are available from the UK Biobank (UKB), which can be accessed via https://www.ukbiobank.ac.uk/, accessed on 16 November 2021.

## Supplementary Materials

The following supporting information can be downloaded at: https://www.mdpi.com/article/10.3390/genes13010037/s1, Figure S1: Manhattan plots of the tissue-specific (TS-TWAS) results from the UKBB cohort. *Y*-axis is the −log10($p_{\mathrm{gs}}$) from S-PrediXcan. An FDR cutoff of 0.05 was shown as a blue line. Genes passing this FDR cutoff were labeled in black color. Table S1: the prevalence (%) of self-reported health conditions among 142,202 UKBB participants with available genotypic and CPD data. Table S2: the frequency of alcohol and cannabis consumption among 142,202 UKBB participants with available genotypic and CPD data. Supplementary file 1: a comparison of brain tissue-specific genes between the associations with nicotine dependence (e.g., CPD) from both the UKBB cohort and GSCAN cohort. Supplementary file 2: full output of pathway enrichment analysis for each our detected meta-pattern category.
