# Supplementary material for: Meta-Analysis of Transcriptome-Wide Association Studies across 13 Brain Tissues Identified Novel Clusters of Genes Associated with Nicotine Addiction
Source: Genes (Basel). 2021 Dec 23;13(1):37. doi: 10.3390/genes13010037 (PMC8775257; doi:10.3390/genes13010037)
Supplement: Supplementary file 1 [file genes-13-00037-s001.zip › Supplementary Figure and Tables.pdf]

genes

Article

Meta-Analysis of Transcriptome-Wide Association Studies across 13 Brain Tissues Identified Novel Clusters of Genes Associated with Nicotine Addiction

Zhenyao Ye [1,2,†], Chen Mo [1,2,†], Hongjie Ke [3,†], Qi Yan [4], Chixiang Chen [2], Peter Kochunov [1], L. Elliot Hong [1], Braxton D. Mitchell [5], Shuo Chen [1,2,*] and Tianzhou Ma [3,*]

[1] Maryland Psychiatric Research Center, Department of Psychiatry, School of Medicine, University of Maryland, Baltimore, MD 21201, USA; zye@som.umaryland.edu (Z.Y.); chen.mo@som.umaryland.edu (C.M.); PKochunov@som.umaryland.edu (P.K.); Ehong@som.umaryland.edu (L.E.H.)
[2] Division of Biostatistics and Bioinformatics, Department of Epidemiology and Public Health, School of Medicine, University of Maryland, Baltimore, MD 21201, USA; Chixiang.Chen@som.umaryland.edu
[3] Department of Epidemiology and Biostatistics, School of Public Health, University of Maryland, College Park, MD 20742, USA; kehj@umd.edu
[4] Irving Medical Center, Department of Obstetrics & Gynecology, Columbia University, New York, NY 10032, USA; qy2253@cumc.columbia.edu
[5] Department of Medicine, University of Maryland School of Medicine, Baltimore, MD 21201, USA; bmitchel@som.umaryland.edu
* Correspondence: shuochen@som.umaryland.edu (S.C.); tma0929@umd.edu (T.M.)
† These authors contributed equally to this work.

Frontal cortex BA9

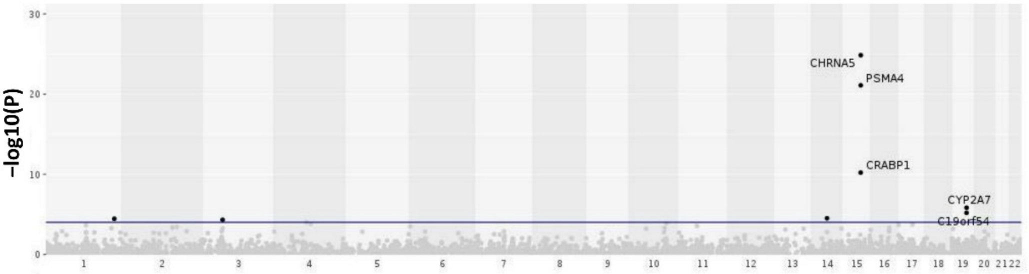

Nucleus accumbens basal ganglia

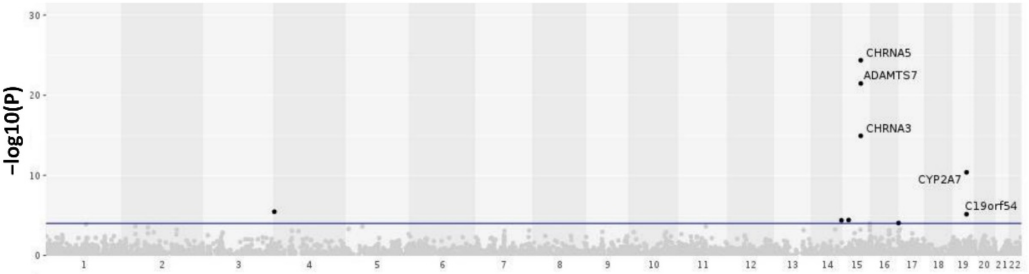

Amygdala

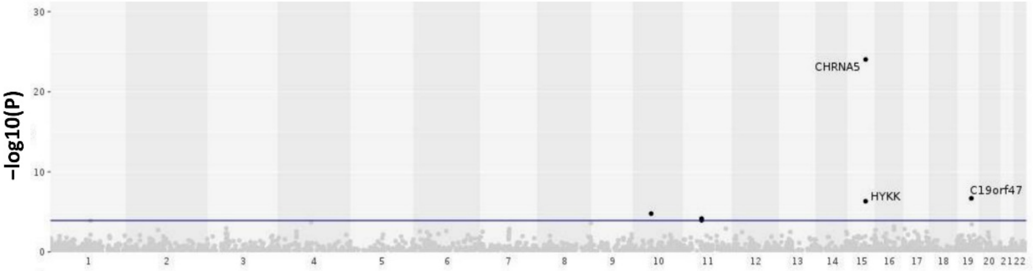

Anterior cingulate cortex BA24

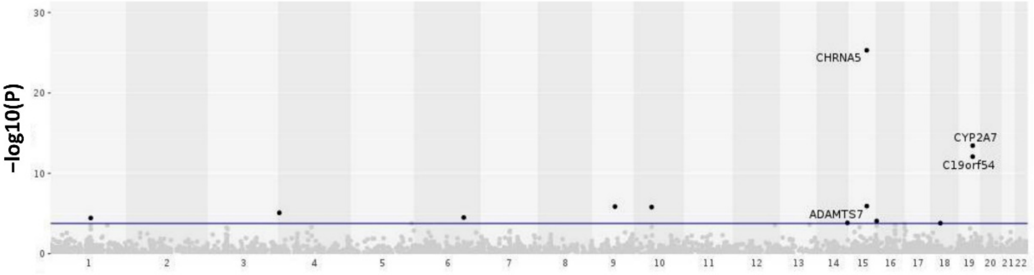

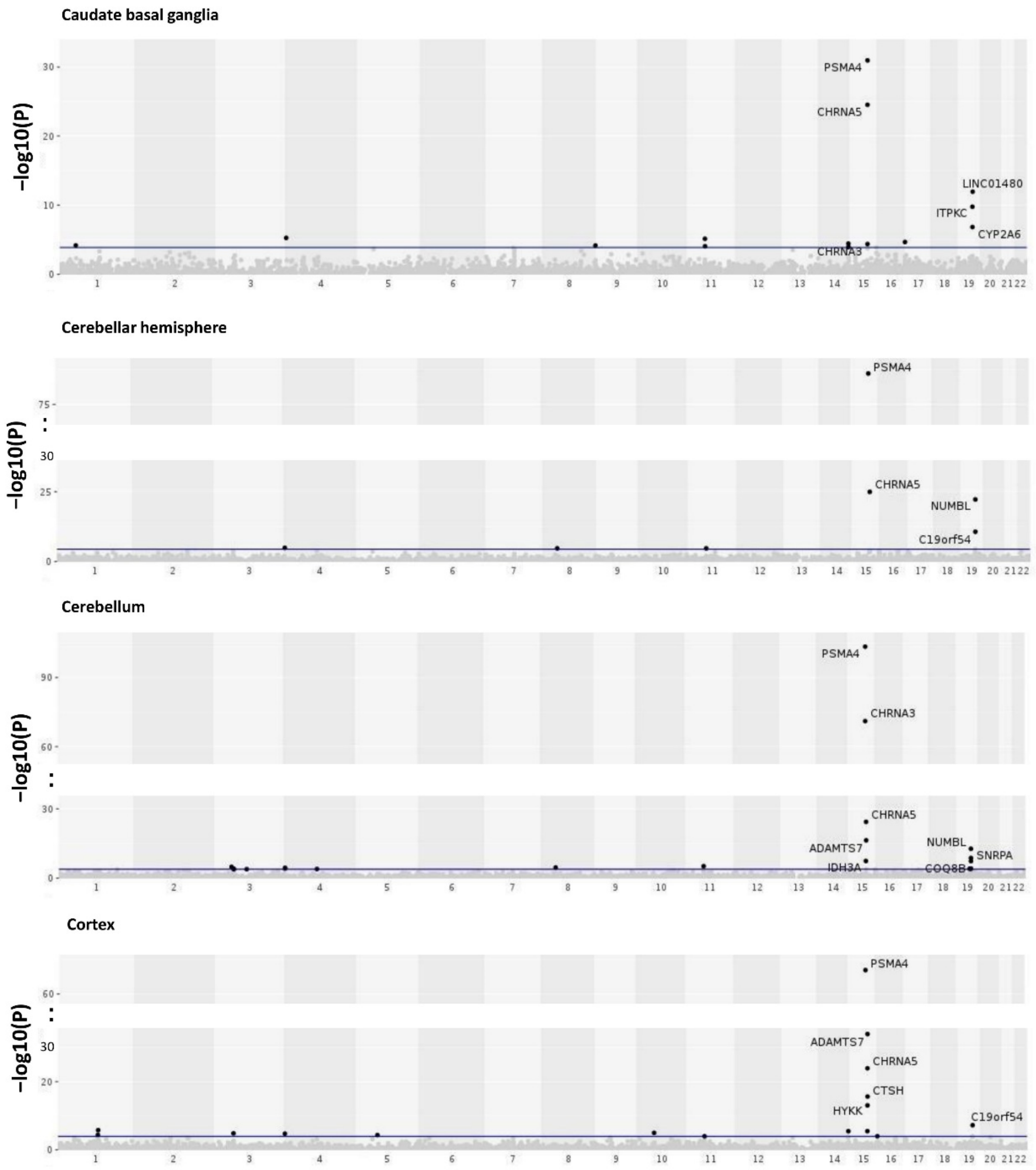
Caudate basal ganglia
−log10(P)
PSMA4
CHRNA5
LINC01480
ITPKC
CYP2A6
CHRNA3
Cerebellar hemisphere
−log10(P)
PSMA4
CHRNA5
NUMBL
C19orf54
Cerebellum
−log10(P)
PSMA4
CHRNA3
CHRNA5
ADAMTS7
NUMBL
SNRPA
IDH3A
COQ8B
Cortex
−log10(P)
PSMA4
ADAMTS7
CHRNA5
CTSH
HYKK
C19orf54

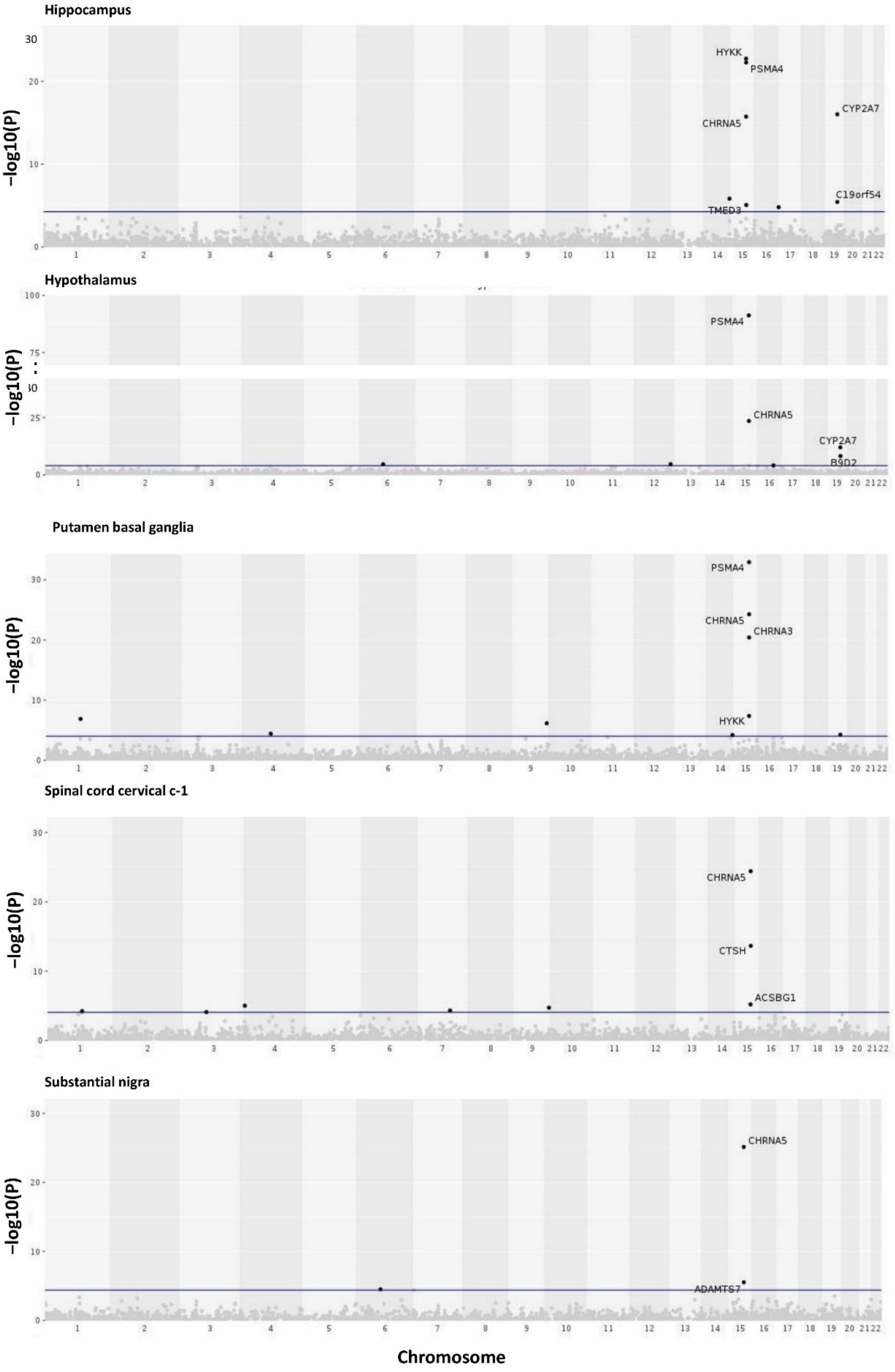
Hippocampus
HYKK
PSMA4
CHRNA5
CYP2A7
TMED3
C19orf54
Hypothalamus
PSMA4
CHRNA5
CYP2A7
B9D2
Putamen basal ganglia
PSMA4
CHRNA5
CHRNA3
HYKK
Spinal cord cervical c-1
CHRNA5
CTSH
ACSBG1
Substantial nigra
CHRNA5
ADAMTS7
−log10(P)
Chromosome

genes

MDPI

Figure S1. Manhattan plots of the tissue-specific transcriptome wide association analysis (TS-TWAS) results from UK Biobank (UKBB) cohort. Y-axis is the –log10(p_{gs}) from S-PrediXcan. A false discovery rate (FDR) cutoff of 0.05 was shown as a blue line. Genes passing this FDR cutoff were labeled in black color.

Table S1. The prevalence (%) of self-reported health conditions among 142,202 UK Bioank (UKBB) participants with available genotypic and cigarette per day (CPD) data.

Self-Reported Disease	Yes	No	Prevalence (%)
	N = 142,202		
viral infections of the CNS	111	142091	0.0781
HIV	71	142131	0.0499
Helminthiases	48	142154	0.0338
malignant neoplasms outside of CNS	21669	120533	15.2382
malignant neoplasms of meninges	8	142194	0.0056
malignant neoplasms of brain	226	141976	0.1589
malignant neoplasms of CNS	13	142189	0.0091
Organic, including symptomatic, mental disorders	1344	140858	0.9451
mental and behavioural disorders due to psychoactive substance use	16164	126038	11.3669
schizophrenia, schizotypal, and delusional disorders	574	141628	0.4037
Bipolar affective disorder	550	141652	0.3868
mental retardation	23	142179	0.0162
disorders of psychological development	160	142042	0.1125
behavioral and emotional disorders with onset usually occurring in childhood and adolescence	119	142083	0.0837
inflammatory disease of CNS	273	141929	0.1920
systemic atrophies primarily affecting CNS	189	142013	0.1329
extrapyramidal and movement disorders	1011	141191	0.7110
other degenerative disease of the nervous system	672	141530	0.4726
demyelinating diseases of the CNS	651	141551	0.4578
episodic and paroxysmal disorders	7157	135045	5.0330
nerve, nerve root, and plexus disorders	8524	133678	5.9943
polyneuropathies and other disorders of the peripheral nervous system	1059	141143	0.7447
disease of myoneuraal junction and muscle	229	141973	0.1610
cerebral palsy and other paralytic syndromes	1184	141018	0.8326
other disorders of the nervous system	2199	140003	1.5464
cerebrovascular diseases	4987	137215	3.5070
Congenital malformations of the nervous system	112	142090	0.0788
abnormal findings on diagnostic imaging of CNS	167	142035	0.1174
abnormal results of function studies of CNS	6	142196	0.0042
injuries to the head	3628	138574	2.5513
injury of cranial nerves	8	142194	0.0056
intracranial injury	610	141592	0.4290
crushing injury of head	3	142199	0.0021
traumatic amputation of part of head	4	142198	0.0028

other and unspecified injuries of head	1006	141196	0.7074
stroke	2092	140110	1.4711
angina pectoris	1842	140360	1.2953
myocardial infarction	2493	139709	1.7531
essential (primary) hypertension	370	141832	0.2602
chronic obstructive pulmonary disease	2080	140122	1.4627
insulin-dependent diabetes mellitus	28	142174	0.0197

Table S2. The frequency of alcohol and cannabis intake among 142,202 UK Biobank (UKBB) participants with available genotypic and cigarette per day (CPD) data.

	Number of participants	Proportion (%) of the total N=142,202
Alcohol intake frequency		
Daily or almost daily	39822	28.0038
Three or four times a week	31986	22.4934
Once or twice a week	32245	22.6755
One to three times a month	13887	9.7657
Special occasions only	14865	10.4534
Never	926	0.6512
N/A	129	0.0907
Maximum frequency of taking cannabis (marijuana, grass, hash, ganja, blow, draw, skunk, weed, spliff, dope)		
Less than once a month	8196	5.7636
Once a month or more, but not every week	1854	1.3038
Once a week or more, but not every day	3091	2.1737
Every day	1637	1.1512
N/A	127424	89.6077
